# Women in neuroscience: Where are we in 2019?

**DOI:** 10.1002/jnr.24570

**Published:** 2020-04-08

**Authors:** Saima Machlovi, Adriana Pero, Sabrina Ng, Margaret Zhong, Dongming Cai

**Affiliations:** ^1^ Department of Neuroscience Icahn School of Medicine at Mount Sinai New York NY USA; ^2^ Department of Neurology Icahn School of Medicine at Mount Sinai New York NY USA; ^3^ Department of Science and Technology Studies Cornell University Ithaca NY USA; ^4^ Department of Neuroscience and Behavior Barnard College of Columbia University New York NY USA; ^5^ Research and Development James J. Peters VA Medical Center Bronx NY USA; ^6^ Neurology Section James J. Peters VA Medical Center Bronx NY USA

Women have traditionally been excluded from positions of power, specifically in the field of science, technology, engineering, and mathematics (STEM). Believed to diminish the intellectual capabilities of scholars, women were historically barred from many opportunities. The first woman was not admitted to the forefront of scientific developments founded in 1666, the French Academy of Sciences until 1979. Over the past half a century, this situation has gradually improved with changes in the scientific landscape and an increased awareness of gender bias in science. More women are encouraged to pursue careers in science, and their interests in science are being cultivated. However, we have not yet reached equality. Where are we in 2019?

Data suggest that female scientists continue to face challenges and hurdles in their career advancement. As to be expected from the inequality of women in STEM, inequality for women also permeates the field of neuroscience. Equal percentages of males and females with college degrees in STEM fields proceed to PhDs (~10%). Similarly, there are equal percentages of males and female PhDs who receive assistant professor job offers (~35%). However, women only comprise one third or less of assistant professors in STEM fields and 7%–18% of full professors (Ceci, Ginther, Kahn, & Williams, 2014; Williams, 2017). In neuroscience, a survey in 2003 indicated that females accounted for 50% of neuroscience PhD students, but only 25% of tenure‐track faculty and 22% of tenured full professors (“Women in neuroscience: A numbers game,” 2006). In 2017–2018, females comprised of 53% of PhD matriculates, 30.8% of tenure‐track faculty but only 13.8% of tenured full professors in academic neurology and neuroscience fields (McDermott et al., 2018; SFN Reports, 2017).

As the data indicate, women make up at least 50% of the class at predoctoral and doctoral phases of their education. However, as women progress through their careers, a large number of women leave the field and the proportion of men in the field and in positions of power sharply increases. One reason for the drop in the proportion of women is the discrepancy in societal obligations between men and women. In addition to keeping up with the demands of the field, women are also expected by society to have and care for children. “At the end of the day you are fighting for a position and if your competitor didn't have to go over this maternity process, he is for sure in a better position that you are,” said Elisa Navarro, a current postdoctoral candidate of the Icahn School of Medicine at Mount Sinai.

Interestingly, evidence suggests that instead of being denied job opportunities in academic science, female scientists tend to choose leaving the field in order to maintain work–life balance. A general belief in our society is that academic careers are not compatible with family life. A 2011 survey by colleagues in UC Berkeley indicated that the ratio of female postdocs who plan to start families or who already had children before taking the postdoctoral positions chose to opt out of academic career paths was much higher than men (Ceci et al., 2014). Balancing life and career is identified as a major hurdle for women scientists. A survey by The American Association for the Advancement of Science (AAAS) in 2011 found that the primary reasons male scientists left academic careers were grants and funding, whereas for female scientists balancing life and career, having and rearing children and gender bias were placed before grants and funding (Baker, 2011).

A more insidious reason for the drop in the number of women especially in positions of power might be subtle biases and stereotypes against women scientists in the workplace. Saima Machlovi, a PhD student of the Icahn School of Medicine at Mount Sinai commented, “This can be observed at seminars. Female presenters are frequently interrupted throughout their talks, and their findings are more easily questioned by others compared to their male colleagues. This may represent an internal bias that women are not capable of making remarkable discoveries however our history proves otherwise.” Examples of men in powerful roles asserting their beliefs that women are incapable of the same scientific work as men can be found throughout history. High school students everywhere learn the names of Watson and Crick, but few recognize the name of Rosalind Franklin. James Watson, one of the discoverers of DNA structures, was known for his racist, sexist, and homophobic beliefs. He not only undermined and belittled his female colleague Rosalind Franklin's contributions to the discovery of DNA structure but also suggested using knowledge of DNA to make all girls pretty. When commenting on the increase in women in science he said, “It would be more fun for the men, but they are probably less effective.” Thankfully, people like Ben Barres, a transgender neuroscientist, are fighting against these misbeliefs against women in science. A personal experience shared by Ben was another example of discrimination. Ben overheard a comment about his talk at a scientific symposium that he did better work than his sister, Barbara Barres, who was actually Ben before he transitioned.

The bias can be manifested in publications as well. One study found that women had to publish 3 more papers in high‐profile journals, or 20 more in less impactful journals, to be considered as productive as their male colleagues when applying for postdoctoral positions (Hill, Corbett, & Rose, 2010). In a study that analyzed neuroscience journals from 2005 to 2017, women were underrepresented in many high‐profile journals, with only 29.8% of all authors being women, 33.1% of first authors being women, and 18.1% of last authors being women (Bendels, Muller, Brueggmann, & Groneberg, 2018; Shen et al., 2018). It was also noted that the number of female authors of a journal was negatively correlated with its impact factor in 5 years. The highest number of female last authors was in Neuropsychology Review (39.04%), and the lowest was in Nature (14.64%). The highest number of female first authors was in Neuropsychology Review (52.58%), and the lowest was in Nature (25.22%). The average rate of increase was less than 1% per year for first female authors and less than 0.5% per year for last female authors (Bendels et al., 2018; Shen et al., 2018).

Moreover, data suggest that women are still not treated as equals to men in many areas of science despite the vast strides that women have made in the last 50 years. For example, a study by Academic Medicine showed a substantial salary difference between male and female scientists by about $20,000 (Girod et al., 2016; Valantine, 2016). Studies by MIT in 1999 showed that female faculty tended to have less space and fewer resources than their male colleagues with similar academic achievements. It was estimated that female scientists got 40% less start‐up money on average than male scientists (Girod et al., 2016; Valantine, 2016). Of the Research Publication Grants awarded, only 33% are awarded to women. Additionally, only 26% of the Research Center Grants are awarded to women. Not only are fewer grants awarded to women but the value of the grants awarded to women are smaller, with the average award being $505,271 for women compared to $579,673 for men (Pohlhaus, Jiang, Wagner, Schaffer, & Pinn, 2011). Studies also show that Asian and African‐American women scientists had a lower chance of receiving funding support, suggesting a double discrimination for women scientists of color (Girod et al., 2016; Valantine, 2016).

Besides gender bias, sexual harassment, a form of sex discrimination, is being increasingly reported by women in science. Sexual harassment can be blatant or can take the form of microaggressions. Microaggressions have been defined as “brief and commonplace daily verbal, behavioral, or environmental indignities, whether intentional or unintentional, that communicate hostile, derogatory, or negative prejudicial sights and insults toward any group, particularly culturally marginalized groups” (Sue et al., 2007). The accumulation of microaggressions over time is believed to lead to lower self‐confidence, which can cause mental health problems and possibly associate with women's leaving science field.

With challenges mentioned above like difficulties in maintaining work–life balances, less control of work environment with a lower likelihood of promotion into leadership positions, confronting biases and stereotypes, as well as experiencing higher rates of sexual harassment, women in science and healthcare professionals have a higher risk of developing burnout. For example, surveys have found a much higher percentage of female physicians’ self‐reported burn out symptoms and dissatisfaction with work–life integration than male physicians (Tawfik et al., 2018; West, Dyrbye, & Shanafelt, 2018). While many factors contributing to burnout among male and female scientists and healthcare professionals are comparable, it is important to recognize certain gender‐based differences in contributing factors of burnout and thereby tailor preventive and interventional strategies to mitigate burnout symptoms and increase retention rate among women scientists (Templeton et al., 2019).

Despite increasing numbers of women in science in decades, the proportion of female scientists in leadership positions remains low. The lack of appropriate representation, compensation, and recognition of women in STEM is appalling, which results in a loss of talent and idea, leading to research from a male perspective. For example, the disparate representation of women in science has led to a prolonged lack of understanding, such as assuming the egg plays a passive role in fertilization (Martin, 1991), and physical harm, such as introducing drugs into the market that have adverse effects for women since they were tested only in men (Zakiniaeiz, Cosgrove, Potenza, & Mazure, 2016). Compelling data have shown that diverse groups including women and people of color generate more creative ideas and improve scientific outcomes than homogenous groups do. For example, papers with more diverse backgrounds of authors received more citations (Jang, 2017).

What can we do better? It is critical to identify barriers that prevent female scientists from career advancement. It is clear that bias and discrimination against women in science has not been adequately addressed. Changes in institutional and departmental structure and culture to develop female faculty value system are important steps to prevent the “leaky pipeline” and improve retention of women in science (Carr et al., 2019). We need mechanisms in place to make success as a woman in science more feasible. When asked about what would make being a woman in science easier, Kathryn Bowles a postdoctoral fellow at Icahn School of Medicine at Mount Sinai commented, “I think the increased inclusion of women and minorities on committees, panels and leadership positions will help resolve some of the biases against women.”

Mentoring and strong leadership to support and promote women's work and sense of voice have been among the most important factors in retaining women scientists in the field. Female role models are particularly critical for recruiting young women scientists and rising stars into the science field and keeping them there. Lack of appropriate role models often leads to feelings of exclusion. Programs such as Maximizing Access to Research Careers, ENDURE Undergraduate Education, and Minority Biomedical Research Support match students with mentors and support a range of activities to increase student involvement and engagement in science. In addition to programs that help students from underrepresented groups, having a mentor is beneficial for all students and especially for students who are underrepresented in a field. Students with mentors perform better academically than students without mentors and women in particular rate the importance of mentorship more highly than men (Fisher, Fried, Goodman, & Germano, 2009). Margaret Zhong, an undergraduate neuroscience major at Barnard College of Columbia University, finds herself in a situation that proves such. “I work in a behavior lab at the Columbia University Medical Center. The entire lab is male, except for the graduate student I work with. Watching her navigate the neuroscience field inspires me to continue on my own path in neuroscience.”

For junior faculty in science, mentorship should focus not only on academic research progresses and grantsmanship training but also on career coaching of leadership skills, effective laboratory management, and conflict resolution, as well as techniques of coping and stress management. The NIH sponsored early career training programs as well as several PI training programs available in academic institutes are good examples. Every female scientist may have a story to tell. Dongming Cai, an Associate Professor of Neurology in Icahn School of Medicine at Mount Sinai, recalled how she wound up a neuroscientist, crediting her parents who nurtured her belief that women can do everything men can, and her science mentors Marie T. Filbin and Paul Greengard were particularly influential, as they encouraged and guided her during her career development while providing supportive environments where women in laboratories were not treated any differently from men.

It is critical to develop transparent mechanisms which allow women scientists to provide feedback that may increase job satisfaction and improve retention of women in science. For example, movements like “MeToo” are bringing awareness to the prevalence of sexual harassment, and are drawing attention to the unequal power dynamic between men and women. Efforts like this are beginning to influence academia, and will eventually lead to more women being in positions of power. In addition, changes at institutional and departmental levels to address concerns and issues about work–life balance and compensation equity among women scientists are important for recruitment and retention of top talent and increased job satisfaction. Examples such as onsite child and elder care, part‐time tenure tracks, and extension of clock for tenure track clock, as well as inclusion of workplace responsibilities like committees and teaching into consideration of promotion processes have been suggested as best practices in this regard (Carr et al., 2019). Other efforts could be beneficial such as offering retention and mentorship grant support by academic institutes and departments in addition to the funding mechanisms provided by federal agencies such as NIH and NSF or institutions like Association for Women in Science. It is also important to recognize that women tend not to ask for salary raises, promotion, or recognition for good work they do. Educating women scientists with better negotiation skills is one way, but more important is to implement a more user‐friendly system to monitor career advancement and job satisfaction of women scientists particularly at early career development stage when most vulnerable to opt out. Establishing a supporting network of women scientists at different academic ranks within the same institute or from different academic institutes to share experiences and provide feedback could be helpful as well.

We need to recognize that even though progress toward increasing women in neuroscience is being made, more still needs to be done. Having a family and furthering a career should not be mutually exclusive. Programs that make maternity leave more accessible or also encourage paternity leave alleviate certain factors that drive women to leave their careers. Additionally, more needs to be done to fight the bias that remains in science. For example, articles submitted for publications or grant applications could be de‐identified for review to better eliminate the possibility of bias. The field of neuroscience is uniquely situated to lead the push for women's equality because of the high numbers of initial interest. We need to work as a team and take steps to foster, encourage, and nurture future women in neuroscience. We should be leaders in the women's fight for equality.

## Conflict of interest

All authors declare that they have no conflict of interest.

## AUTHOR CONTRIBUTIONS

*Conceptualization*, S.M. and D.C.; *Data Curation*, S.M., A.P., S.N. and D.C.; *Formal Analysis*, S.M., A.P., S.N., M.Z., and D.C.; *Methodology*, S.M., A.P., S.N., M.Z., and D.C.; *Investigation*, S.M., A.P., S.N., M.Z., and D.C.; *Validation*, S.M., A.P., S.N., M.Z., and D.C.; *Writing ‐ Original Draft*, S.M., A.P., and D.C.; *Writing ‐ Review & Editing*, S.M., A.P., S.N., M.Z., and D.C.; *Funding Acquisition*, D.C.; *Project Administration*, D.C.; *Resources*, D.C.; *Supervision*, D.C.
